# (η^8^-Cyclooctatetraene)(η^5^-fluorenyl)titanium: a processable molecular spin qubit with optimized control of the molecule–substrate interface†

**DOI:** 10.1039/d4sc03290j

**Published:** 2024-08-09

**Authors:** Sarita Wisbeck, Andrea Luigi Sorrentino, Francielli S. Santana, Luana C. de Camargo, Ronny R. Ribeiro, Enrico Salvadori, Mario Chiesa, Niccolò Giaconi, Andrea Caneschi, Matteo Mannini, Lorenzo Poggini, Matteo Briganti, Giulia Serrano, Jaísa F. Soares, Roberta Sessoli

**Affiliations:** a Department of Chemistry, Federal University of Paraná, Centro Politécnico Jardim das Américas 81530-900 Curitiba PR Brazil; b Department of Chemistry “U. Schiff” (DICUS) and INSTM Research Unit, University of Florence Via della Lastruccia 3-13 50019 Sesto Fiorentino Italy roberta.sessoli@unifi.it; c Department of Chemistry, University of Turin Via Giuria 7 10125 Torino Italy; d Department of Industrial Engineering (DIEF) and INSTM Research Unit, University of Florence Via di S. Marta 3 50139 Firenze Italy; e Institute for Chemistry of OrganoMetallic Compounds (ICCOM-CNR) Via Madonna del Piano 50019 Sesto Fiorentino Italy

## Abstract

Depositing single paramagnetic molecules on surfaces for sensing and quantum computing applications requires subtle topological control. To overcome issues that are often encountered with sandwich metal complexes, we exploit here the low symmetry architecture and suitable vaporability of mixed-sandwich [FluTi(cot)], Flu = fluorenyl, cot = cyclooctatetraene, to drive submonolayer coverage and select an adsorption configuration that preserves the spin of molecules deposited on Au(111). Electron paramagnetic resonance spectroscopy and *ab initio* quantum computation evidence a d_*z*^2^_ ground state that protects the spin from phonon-induced relaxation. Additionally, computed and measured spin coherence times exceed 10 μs despite the molecules being rich in hydrogen. A thorough submonolayer investigation by scanning tunneling microscopy, X-ray photoelectron and absorption spectrocopies and X-ray magnetic circular dichroism measurements supported by DFT calculations reveals that the most stable configuration, with the fluorenyl in contact with the metal surface, prevents titanium(iii) oxidation and spin delocalization to the surface. This is a necessary condition for single molecular spin qubit addressing on surfaces.

## Abstract 2

A comprehensive investigation shows that the replacement of a cyclopentadienyl ligand with fluorenyl increases the adsorption energy of the [FluTi(cot)] spin-qubit candidate on Au(111) without loss of spin density toward the surface.

## Graphical abstract

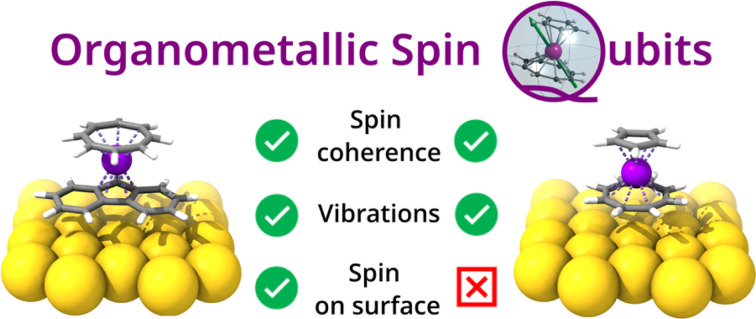

## Introduction

Organometallic sandwich complexes featuring paramagnetic metal ions have garnered significant interest due to their potential in the design of highly efficient single-molecule magnets. This is attributed to the distinct axial symmetry observed in the metal environment.^1,2^ In addition, the polyhapticity of the planar ligands reduces the efficiency of low-energy molecular vibrations in promoting magnetic relaxation.^3,4^ If we consider quantum applications, remarkable electron spin coherence times (above 30 μs at 4.5 K) have been observed for [CpTi(cot)] (Cp^−^ = η^5^-C_5_H_5_^−^ and cot^2−^ = η^8^-C_8_H_8_^2−^), despite the abundant hydrogen environment,^5^ which is a significant source of decoherence. Titanium(iii) sandwich complexes have also been inserted in two-qubit molecular architectures, enabling spectral addressability.^6^ Neutral sandwich complexes are very volatile, allowing the realization of ultra-clean interfaces by sublimation under ultra-high vacuum (UHV) that can be deeply investigated by scanning tunnel microscopy (STM).^7^ They can be used to functionalize STM tips, imparting spin sensitivity at the atomic scale.^8,9^

We successfully deposited [CpTi(cot)] molecules on Au(111) but have also evidenced the difficulty in controlling this interface. Two primary deposition geometries of the molecule were observed due to very similar adsorption energies. More importantly, for the configuration where the cot^2−^ ligand is in contact with the gold, electron transfer occurs from the titanium with a loss of spin density to the surface.^10^ These observations sparked interest in exploring structurally related organometallic complexes endowed with other cyclic polyene ligands that could influence molecule-surface interaction. To achieve improved control of the spin functionalities on the surface, we replaced the cyclopentadienyl with the larger fluorenyl ligand (Flu^−^ = C_13_H_9_^−^) to obtain [FluTi(cot)]. In this case, the more extensive contact area exposed by Flu^−^ to the surface increased adsorption energy without incurring the electronic hybridization, observed for [CpTi(cot)], mediated by the cot^2−^ ligand. In addition, the [FluTi(cot)] higher molecular weight and lower vapor pressure enabled better control over sublimation kinetics.

The synthesis of the complex was performed through a new and accessible methodology that allowed for the isolation of single crystals whose X-ray molecular and crystal structure is described here for the first time. Pulsed electron paramagnetic resonance (EPR) spectroscopy revealed that [FluTi(cot)] retains remarkable coherence times despite the increased number of hydrogen atoms in the pentahapto Flu^−^ ligand. Ultra-thin films down to the monolayer were deposited on Au(111) and investigated *in situ* by STM and X-ray photoelectron spectroscopy. DFT calculations indicated that the adsorption occurs through the fluorenyl ligand and preserves the spin of the titanium ion, which was experimentally confirmed by synchrotron-based investigation.

## Results and discussion

### Synthesis and crystal structure

Fluorene (C_13_H_10_) was deprotonated with *n*-butyllithium, and the orange fluorenyl lithium (LiFlu) suspension was used *in situ* without filtration. The reduction of cyclooctatetraene to cot^2−^ was performed separately with an excess of *n*-butyllithium and the resulting solution was added to the bright yellow suspension of the titanium(iv) precursor, [TiCl_4_(thf)_2_]. Only after this step, the LiFlu suspension was added; this strategy prevented the unwanted oxidation of Flu^−^ by titanium(iv), forming 9,9′-bifluorenyl (Fig. S1†) and a titanium(iii)-thf adduct. See ESI† for more details.

**Fig. 1:**
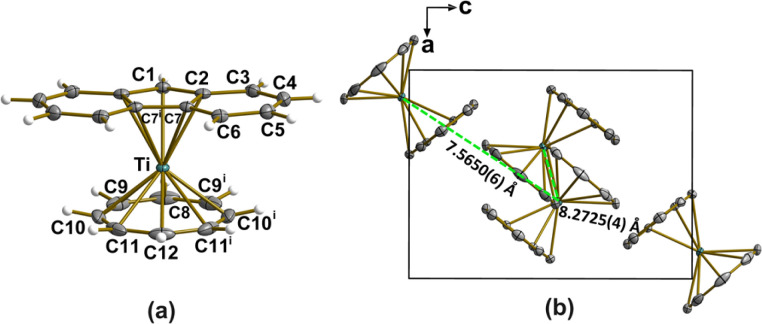
(a) Structural representation of [FluTi(cot)], with the atom numbering scheme. Thermal ellipsoids encompass 50% of the electron density probability. (b) Unit cell representation down the crystallographic *b* axis. Symmetry code (i): *x*, −*y* + 1/2, *z*.

The structure of [FluTi(cot)] was confirmed by single-crystal X-ray diffraction analysis at 100(2) K (Fig. 1, Tables S1 and S2†), which also provided accurate geometrical parameters for the electronic and vibrational theoretical analysis. Data were also collected at room temperature, and the structure was solved (see Tables S1 and S3†). No evidence of the structural transition observed in [CpTi(cot)] was detected,^11^ and the results are not further discussed here as all other investigations were performed at cryogenic temperatures. The complex crystallizes in the orthorhombic *Pnma* space group, with four molecules in the unit cell. Crystallographic *ac* mirror planes pass through Ti, C1, C8, C12, and the middle point of the C7–C7^i^ bond, similar to what happens in [CpTi(cot)]. The two ligand rings are closer to parallel in [FluTi(cot)] (dihedral angle 1.71°) than in [CpTi(cot)] (2.93°) and [CpTi(cht)] (cht = cycloheptatrienyl, 2.55°) measured at the same temperature.^5^ The planar cot^2−^ ring is closer to the titanium(iii) center than the 5-membered ring in the Flu^−^ moiety, consistent with the higher-negative-charge ligand interacting more strongly with the metal. This Ti⋯cot^2−^ distance is marginally smaller in [FluTi(cot)] than in [CpTi(cot)] (1.420 *versus* 1.442 Å), while the distance to the Flu^−^ plane increases slightly compared to the Ti⋯Cp^−^ separation in [CpTi(cot)] (2.056 and 2.028 Å, respectively). In the crystal, the molecules show the typical T-packing of homo- and heteroleptic sandwich complexes (Fig. 1b), though with a larger deviation from orthogonality (79.07° *vs.* 84.38° for [CpTi(cot)] at 100 K).^5^

### Electronic structure

**Fig. 2:**
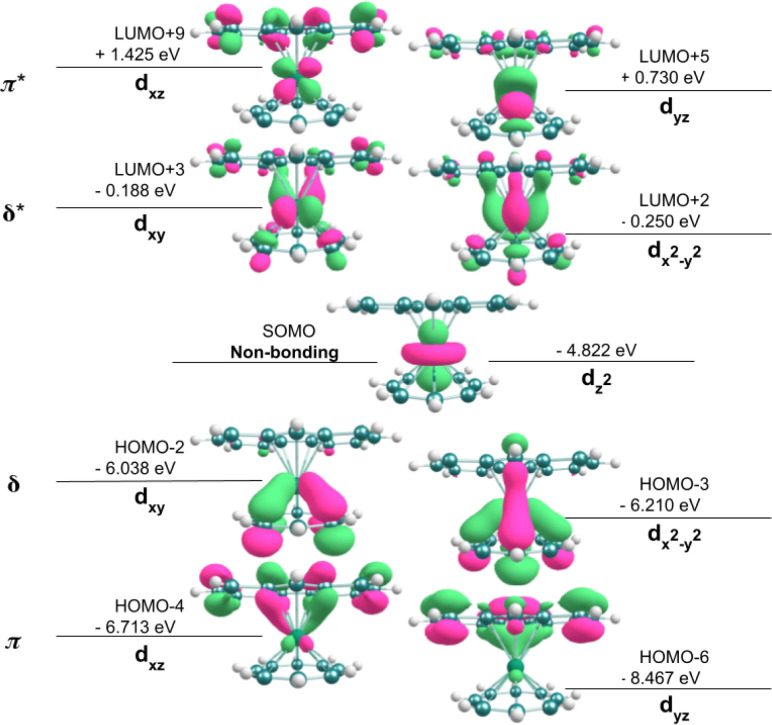
Energies and contour surfaces (50%) of frontier molecular orbitals in [FluTi(cot)] (spin α).

DFT calculations were performed (see Experimental section) to better understand the electronic structure and reactivity of [FluTi(cot)], especially the differences as compared to [CpTi(cot)].^5^ Similar to the latter, [FluTi(cot)] exhibits the non-bonding d_*z*^2^_ as the magnetic orbital (SOMO), as shown in Fig. 2. Moreover, in [FluTi(cot)], the metal d_*x*^2^−*y*^2^_ and d_*xy*_ orbitals selectively interact with the cyclooctatetraene (cot^2−^) ring for δ-bond formation, while the d_*xz*_ and d_*yz*_ orbitals participate in π-bonding with the fluorenyl ligand. As expected, two bonding combinations (ligand-based) and two antibonding ones (metal-based) result from the interaction. However, the anionic fluorenyl ligand has *C*_2v_ point group symmetry, with non-degenerate π orbitals (Fig. S2†). For this reason, the two molecular orbitals of each combination (π, δ, π*, and δ*) shown in Fig. 2 are also non-degenerate.

### EPR spectroscopy

**Fig. 3:**
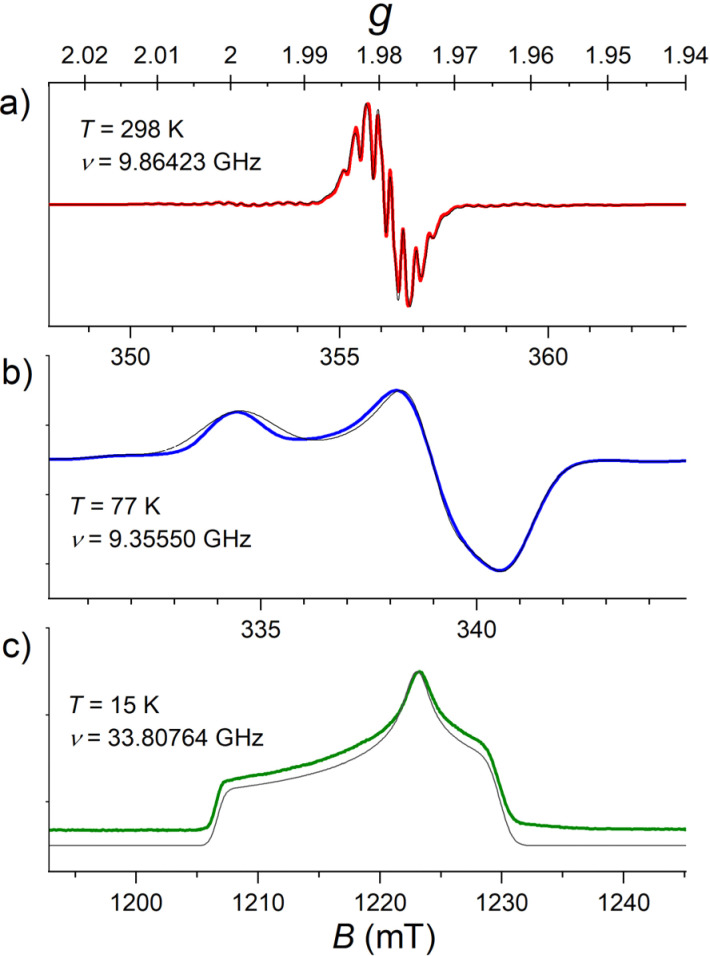
(a and b) X-band CW EPR spectra recorded for a 1.0 mmol L^−1^ solution of [FluTi(cot)] in toluene at two temperatures (298 K and 77 K); (c) Q-band echo-detected spectrum of a 0.5 mmol L^−1^ solution in toluene-d_8_ at *T* = 15 K. Simulated spectra are shown as thin black or grey lines. A common *g* scale is reported on the top to facilitate the comparison.

The X-band CW EPR spectra of a 1.0 mmol L^−1^ solution of [FluTi(cot)] in toluene at 77 K and room temperature are shown in Fig. 3, while intermediate temperatures are available in Fig. S3.† In the frozen solution spectrum recorded at 77 K (Fig. 3b), the *g* tensor components agree with literature values (1.966/1.975/2.000 respectively)^12^ and evidence a rhombic species, differently from the axial [CpTi(cot)]. This comes from the lower symmetry of the fluorenyl ligand compared to the Cp^−^. The *g*_*z*_ component, very close to the free electron *g*_e_ value (2.0023), is clear evidence for the unpaired electron occupying a d_*z*^2^_ orbital, in line with the results of molecular orbital calculations. The weak lines generated by the hyperfine interactions with ^47^Ti (*I* = 5/2) and ^49^Ti (*I* = 7/2) were simulated considering the low natural abundances of 7.44% and 5.41%, respectively. The A values obtained both at 77 K and room temperature reported in Table 1 also comply with an earlier literature report (*A*_iso(Ti)_ = 39.5 MHz *vs.* 41.0 MHz).^12^

**Table 1:** Experimental and computed spin Hamiltonian parameters. The former were obtained from simulation of CW X-band spectra. Hyperfine couplings are in MHz

	298 K	77 K	Calculated
*g*_*x*_		1.9635	1.9639
*g*_*y*_		1.9740	1.9724
*g*_*z*_		2.0005	2.0019
*g*_iso_	1.9788		1.9794
*A* ^Ti^_*x*_		56.15	58.17
*A* ^Ti^_*y*_		53.54	55.16
*A* ^Ti^_*z*_		13.89	4.05
*A* ^Ti^_iso_	39.49		39.126
〈*A*^Hcot^〉	8.62		7.54
*A* ^HFlu1^	5.35		4.1961
〈*A*^HFlu2^〉	—		0.64

The room temperature spectrum, Fig. 3a, evidences the unpaired electron super-hyperfine interactions with the hydrogen nuclear spins in the cot^2−^ and fluorenyl rings. Unlike the reported spectra of [CpTi(cot)],^5^ where distinct superhyperfine lines were only observed at *ca.* −30 °C,^13^ spectral hyperfine and superhyperfine features were resolved for [FluTi(cot)] at room temperature. This is assigned to the considerably slower rotation of the heavier fluorenyl ring compared to the cyclopentadienyl ligand in [CpTi(cot)].^5,13^ The super-hyperfine couplings extracted from the simulation are *a*_iso_*H*_(cot)_ = 8.62 MHz, *a*_iso_*H*^(1)^_(Flu)_ = 5.35 MHz, see Table 1, comparable to those found in the literature.^12^ No clear evidence of the coupling to the hydrogen atoms of the two benzo-substituents of the fluorenyl ligand was observed, in agreement with the longer Ti⋯H distances: in the C_5_ ring, 2.979 Å (1H), and in C_6_, average 3.882 Å (2 × 2H) and 4.876 Å (2 × 2H).

To better rationalize the EPR results, we computed the spin Hamiltonian parameters by DFT, and the most relevant results are listed in Table 1. The *g*-tensor, the ^57^Ti hyperfine, and *H*_(cot)_ super-hyperfine couplings are in very nice agreement with the simulated values, which are also like those of [CpTi(cot)], confirming the negligible effect of the two benzo-substituents on the electronic and magnetic properties of the metal ion. The large coupling found for *H*_(cot),_ as in [CpTi(cot)], arises from the small σ interaction between the d_*z*^2^_ orbital and the π system of the cot^2−^ ligand. The nine *H*_(Flu)_ nuclei, in turn, can instead be divided into two different classes: (i) the strongly coupled proton of the central C_5_ ring, whose hyperfine coupling has a large Fermi-contact contribution and is comparable to the Cp's H nuclei in [CpTi(cot)]; (ii) the other eight protons of the fused benzene rings, whose isotropic coupling is one order of magnitude lower (see Table S4† for individual couplings). The computed super-hyperfine couplings indicate that the contact contribution dominates over the dipolar one for the hydrogen atoms of the benzo groups. In fact, one of the largest interaction is found with one of the most distant H atoms (bound to C4 in Fig. 1) and its symmetry-related analog.

**Fig. 4:**
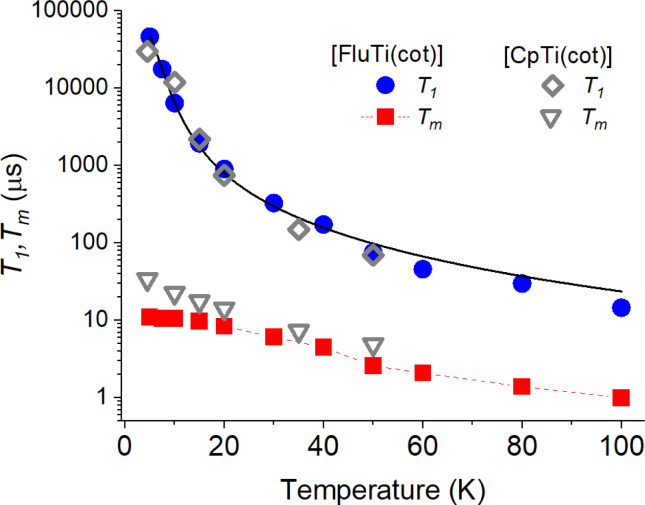
Temperature dependence of the *T*_1_ and *T*_*m*_ of a 0.5 mmol L^−1^ solution of [FluTi(cot)] in toluene-d_8_, superimposed to those of [CpTi(cot)] we reported in ref. 5. The black solid line corresponds to the computed (*T*_1_) values (see text).

Pulsed EPR techniques were used to investigate the spin dynamics of [FluTi(cot)] in a 0.5 mmol L^−1^ solution in toluene-d_8_. Fig. 3c shows the echo-detected Q-band spectrum at 15 K with its simulation. The *g* parameters used in the simulation (*g*_*x*_ = 1.964, *g*_*y*_ = 1.974, *g*_*z*_ = 2.0018) nicely match those employed to simulate the CW spectrum (Table 1). Both *T*_1_ and *T*_*m*_ measurements were performed at a magnetic field corresponding to the highest echo intensity (*B* = 1223.2 mT). Coherent spin manipulation was confirmed by nutation experiments, where the extracted frequency scales linearly with the *B*_1_ amplitude (see Fig. S4†). *T*_1_ measurements were performed using the inversion recovery pulse sequence; the experimental traces were fit with stretched exponential functions, and the stretching parameter ranged from 0.70 to 0.90. The temperature dependence of the extracted *T*_1_ values is shown in Fig. 4, and the best-fit curve was obtained assuming that low-energy optical vibrations are responsible for the Raman-like process:^14,15^1
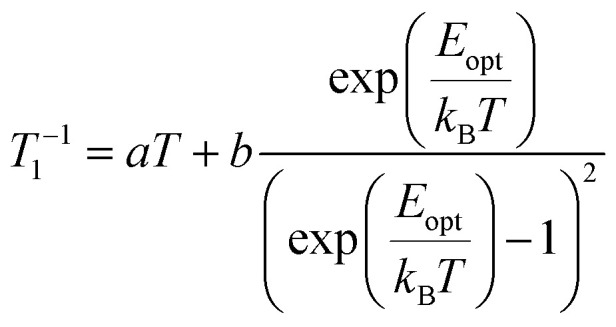


The best-fit parameters are *a* = 4.01 ± 0.02 s^−1^, *b* = 7600 ± 600 s^−1^, and *E*_opt_ = 29.5 ± 0.5 cm^−1^. These values do not differ significantly from those of [CpTi(cot)] obtained in the same conditions, *e.g.*, *E*_opt_ 32 cm^−1^ for the latter, in agreement with the very similar behavior of the two compounds (see Fig. 4).

To better understand this somehow unexpected similarity, the vibrational properties of [FluTi(cot)] were computed in the gas phase considering the interaction with the solvent through the implicit model.^16^ The results are reported in Table S5.† The lowest optical mode is computed at 31.5 cm^−1^, thus in agreement with the estimation from the *T*_1_ fitting. This vibration corresponds to an opposite rotation of the two planar ligands (see Table S6† for a pictorial view of the normal modes), weakly affecting the overlap of the metal and ligand orbitals. Interestingly, at variance with [CpTi(cot)], the solvent does not alter the energy of this vibration. The next vibrations are significantly higher in energy, above 80 cm^−1^, and do not impact the experimental data in the investigated temperature range.

Experimental *T*_*m*_ values were extracted by fitting the experimental decay traces using a stretched exponential function. For [FluTi(cot)], *T*_*m*_ levels off at low temperatures and reaches *ca.* 11 μs at 5 K, a value three times shorter than the observed for [CpTi(cot)] (Fig. 4). Differences in phonon-induced decoherence can be safely excluded, given the two molecules' similar low-energy vibrational spectrum. Indeed, the *T*_*m*_ values of the two compounds tend to converge at increasing temperatures, where phonons have a greater influence.

To rationalize the origin of this difference, we estimated the coherence time, assuming that instantaneous diffusion is the primary decoherence process (see ESI, page S8†).^17^ This approach provided *T*_*m*_ values of 58 μs and 44 μs for [CpTi(cot)]^5^ and [FluTi(cot)], respectively. For the latter, the experimental value is four times lower than the computed one. The more pronounced decrease in *T*_*m*_ can be safely ascribed to the presence of eight weakly coupled hydrogen atoms from the benzo groups (in the fluorenyl ligand) compared to the five equally and strongly-coupled hydrogen atoms of the Cp ligand in [CpTi(cot)]. The increased distance between the benzo hydrogen atoms and the paramagnetic center places them on the edge of the frozen sphere, decreasing the protection of the Ti unpaired electron from spin diffusion decoherence mechanisms.^18^

### Surface deposition and characterization

**Fig. 5:**
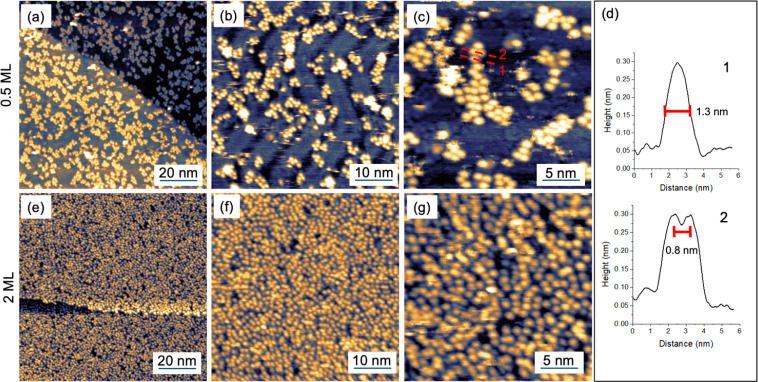
STM images (*T* = 35 K) of a submonolayer (a–c) and a 2 ML deposit (e–g) of [FluTi(cot)] on Au(111). (d) Line profiles taken across the red dashed lines in panel c.

[FluTi(cot)] was deposited by sublimation at 385 K in UHV (with a base pressure of 10^−8^ mbar) on a Au(111) single crystal. A sub-monolayer (ML) amount was deposited by exposing Au(111) to the molecular flux for 20 min, and the surface coverage was checked *in situ* by Scanning Tunnel Microscopy (STM), see Fig. 5a–c. Coverage of about 50% of the surface (0.5 ML) was observed by STM images at a large scale, as reported in Fig. 5a. In panel Fig. 5b, the herringbone reconstruction typical of the Au(111) surface is fairly visible within the molecular layer, though the high mobility of the molecules hinders a good resolution of the underlying substrate even at low temperatures (35 K). Such mobility indicates a weak interaction with the substrate, in agreement with the DFT calculations (see below). At higher magnifications (Fig. 5c), single molecules appear as round, bright features on the gold surface without a preferential order.

The absence of an ordered molecular layer agrees with the low symmetry of [FluTi(cot)], which hinders long-range dense packing in a bidimensional lattice. The line profile of a single and two adjacent molecular units (dashed lines in Fig. 5c) are shown in panel 5d. The single feature, as seen in line profile 1, shows a lateral dimension of about 1.3 nm in line with the STM simulation by DFT (see below), while the molecule–molecule distance estimated by line profile 2 is about 0.8 nm. The latter value is comparable to the distances between nearest neighbor molecules in the crystal, which are approximately 0.76–0.83 nm (see Fig. 1b).

Fig. 5e–g show STM images for a 2 ML sample (obtained by increasing the deposition time to 70 min) at the equivalent scales of the 0.5 ML sample. In agreement with the sub-monolayer observations, molecules pack without evidence of order and with an average molecule–molecule distance of about 0.8 nm.

**Fig. 6:**
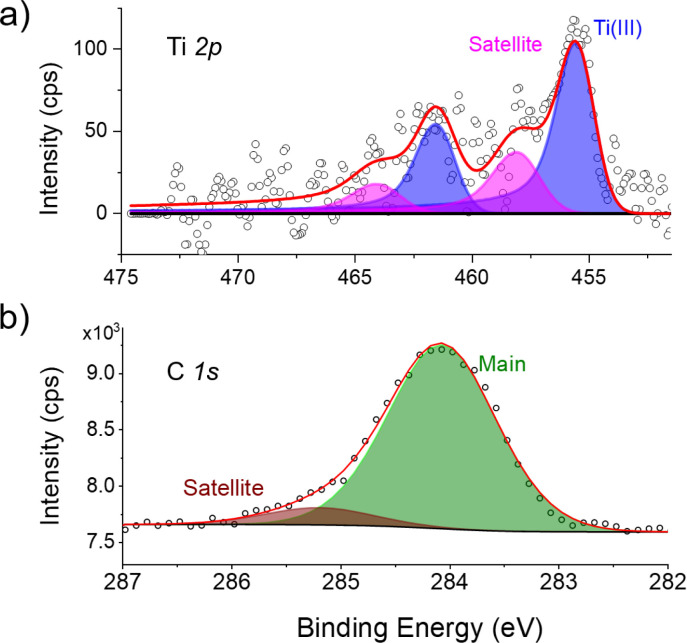
Ti 2p (a) and C 1s (b) XPS spectra of [FluTi(cot)] deposited on Au(111) as a sub-monolayer. Peak color code: blue, Ti^III^ main peaks; magenta, Ti^III^ satellite peaks; green, C 1s main component; brown, C 1s satellite/shake-up component.

The submonolayer sample was investigated by X-ray photoemission and absorption spectroscopies to characterize the magnetic and chemical properties of the compound in contact with the surface. Fig. 6 shows the X-ray photoelectron spectroscopy (XPS) spectra of the Ti 2p (a) and C 1s (b) core-level regions. Clearly, the different noise levels of the C 1s and Ti 2p XPS regions are due to the low amount of Ti centers in the [FluTi(cot)] submonolayer and the low Ti/C ratio (1 : 21) in the molecular structure. In Fig. 6a, the main Ti 2p_3/2_ peak and the corresponding Ti 2p_1/2_ spin–orbit (SO) component are visible at 455.4 and 461.4 eV respectively, *i.e.*, 6 eV away from one another.^19^ Related satellite contributions (pink component) appear at higher energies and are ascribed to the presence of paramagnetic Ti^III^ centers in an organometallic environment, thus excluding any redox alteration of the molecules.^20–22^ The XPS C 1s core level spectrum (Fig. 6b) comprises one component at 284.0 eV and a weak satellite peak, ascribable to the aromatic carbon atoms of the molecular backbone, with a Ti/C ratio in agreement with the stoichiometry of the molecule. Interestingly, in [CpTi(cot)], two distinct features in the C 1s region were observed,^10^ which were attributed to an electron transfer from the molecules lying on the cot^2−^ ligand towards the surface, triggered by the presence of neighboring molecules with alternating adsorption geometry.^10^ Thus, the XPS spectra of [FluTi(cot)] reported above to exclude such a selective oxidation process.

**Fig. 7:**
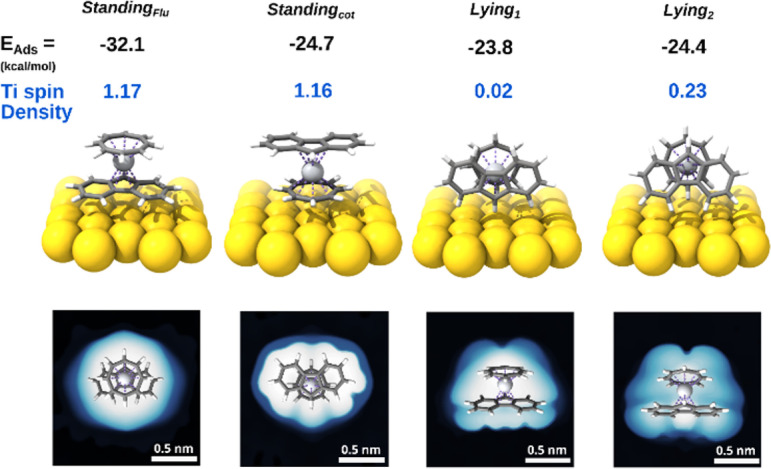
Results of the pDFT calculations: optimized geometries, adsorption energies, Ti spin density, and simulated STM images at −2 V bias (filled states).

To support our hypothesis and rationalize the different behavior of [FluTi(cot)] on the surface, we performed periodic DFT, pDFT, calculations (see Computational details) to simulate the adsorption process of [FluTi(cot)] on Au(111). The molecule can adsorb in four different configurations: two standing orientations, with the cot^2−^ or Flu^−^ rings in contact with the surface, and two *lying* ones, where the molecular axes are almost parallel to the surface (see Fig. 7 for the labeling). The *lying_1_* configuration presents the pentahapto ring of the Flu^−^ ligand in closer contact with the surface than *lying_2_*. All configurations were optimized by pDFT. When the molecule stands on the fluorenyl ligand (*standing*_*Flu*_ orientation), the adsorption energy is stabilized by more than 7 kcal mol^−1^ compared to the other possibilities, which, in turn, differ by less than 1 kcal mol^−1^. Interestingly, the magnitude of the interaction of the 13 fluorenyl sp^2^ carbons with the Au(111) surface is increased by 12.8 kcal mol^−1^ compared to the analogous *standing*_*Cp*_ orientation in [CpTi(cot)]. To investigate adsorption energy dependence on the chosen functional, we performed optimization with two other GGA/meta-GGA density functionals: PBE in its original parametrization^23^ and TPSS.^24^ The results are reported in Table S8.† In both cases, we observe that the *standing*_*Flu*_ geometry is the most stable.

The simulated STM images for the different molecular orientations on Au(111) (Fig. 7) show that the experimentally detected, regular round spots are only expected for the *standing*_*Flu*_ orientation, which is also compatible with the apparent width of 1.3 nm (Fig. 5d). For *standing*_*cot*_, an oval/elliptical shape should be observed, while an irregular appearance is expected for the *lying* arrangements. Computed adsorption energies and simulated STM images allow us to infer that [FluTi(cot)] adsorbs on Au(111) mainly with the fluorenyl ligand in contact with the surface.

Notably, the charge and spin densities of the Ti atom on the Au surface are fully preserved for the most stable *standing*_*Flu*_ configuration (Fig. 7), in agreement with the XPS results. On the other hand, we observe spin delocalization on the Au surface for the two *lying* orientations at the level of the isolated molecule. For *lying_1_* the unpaired electron is completely delocalized on the surface, while it is partially preserved on the Ti atom for *lying_2_*. This effect seems quite robust as it is computed when different Hubbard's *U* values and adsorption sites are employed (see Table S7† for *lying_1_* in two sites). The lack of experimental evidence of titanium oxidation is in line with the less favorable adsorption energies of the *lying* molecules.

**Fig. 8:**
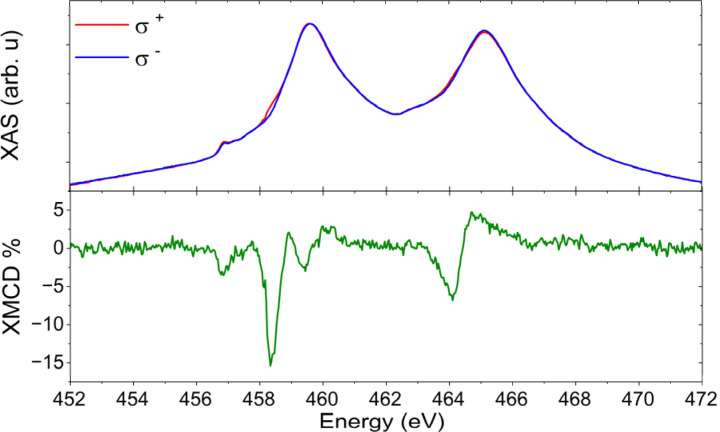
XAS (top) and XMCD (bottom) spectra recorded at *θ* = 0°, *B* = 6 T, and *T* = 2.0 K on a sub-monolayer of [FluTi(cot)] on Au(111) surface.

EPR spectroscopy could detect the persistence of the unpaired spin on surface, but only very recently have the first steps toward developing a spectrometer operating in UHV conditions been reported.^25^ However, the oxidation state and spin density of the titanium ion were unambiguously determined by synchrotron experiments on the [FluTi(cot)] sub-monolayer. X-ray Absorption Spectroscopy (XAS) was used to investigate the Ti L_2,3_ edges, as presented in Fig. 8. The observed lineshape agrees with previous reports for Ti^III^ in different materials.^26–29^ The magnetic properties of the monolayer were then addressed by measuring the signals with right (σ^−^) and left (σ^+^) circularly-polarized X-rays and evaluating the XMCD (X-ray Magnetic Circular Dichroism) data as (σ^−^ – σ^+^). The XMCD signal reported in Fig. 8 was measured at normal incidence, *θ* = 0°, under an applied field of 6 T, and reveals a main dichroic peak at 458.3 eV. XMCD investigations of organometallic Ti^III^ systems are unprecedented and do not allow a direct comparison. Still, both the energy and shape of our XMCD spectrum are comparable to those of photoreduced TiO_2_.^30^ As a matter of fact, the evidence of a dichroic signal at the Ti edge confirms the presence of a significant fraction of molecules whose adsorption configurations retain the unpaired spin on the early transition metal ion.

## Conclusions

In our search for a highly coherent neutral molecule with *S* = 1/2, suitable for surface deposition and single spin addressing by scanning tunnel microscopy, we first evidenced a complex adsorption scenario for the heteroleptic sandwich complex [CpTi(cot)], with partial loss of the spin density on the Ti atom.

In this thorough investigation, we identified [FluTi(cot)] as a more promising candidate. The replacement of Cp with the bulkier fluorenyl ligand does not affect the computed low-energy vibrational properties, and similar spin-lattice relaxation rates are observed in pulsed EPR experiments. The increased number of protons and, more importantly, their weaker coupling with the titanium(iii) spin slightly reduce the spin coherence at low temperatures. This, however, still exceeds that of other molecular qubits employed for surface deposition, such as phthalocyanine or porphyrin complexes of copper^17^ and vanadyl.^31^

The deposition of [FluTi(cot)] on a noble metal surface, studied by STM and photoelectron spectroscopy, shows that the fluorenyl ligand determines only one adsorption orientation for the sandwich molecule thanks to the increased interaction of the fluorenyl with the surface. This disrupts the typical alternating T-shaped packing that characterizes metallocenes^32^ and [CpTi(cot)]^10^ monolayers. Even if DFT spin delocalization appears unrelated to the adsorption energy, the most stable configuration preserves the spin of the Ti ions at the molecule-metal interface, as confirmed by XMCD spectroscopy, making [FluTi(cot)] a very promising candidate for single spin experiments on the surface. The remarkable progress in single spin control by combining STM with microwaves^33,34^ has reached the level of operation of multiqubit atomic platforms,^35^ opening promising perspectives for molecular spin qubits in quantum coherent nanoscience.^36^ The approach to finely tune the molecule-surface interface while keeping control of the magnetic features could also be extended to lanthanide organometallic sandwich molecules that display a wide variety of interesting magnetic phenomena.^37^

## Experimental

### Synthesis

All syntheses were performed in an inert atmosphere (N_2_ 99.999%, Praxair or Air Liquide) employing Schlenk and glove box techniques. Solvents (Honeywell, Aldrich, or Merck) were dried by standard techniques^38^ and freshly distilled under N_2_ before use. Titanium tetrachloride, TiCl_4_, was supplied by Merck and used without further purification. Cyclooctatetraene (cot), provided by Aldrich, underwent five freeze–vacuum–thaw cycles, and was stored in the glove box on previously activated 4 Å molecular sieves. Fluorene (Aldrich), in turn, was recrystallized by dissolving in dry hexane under reflux, then cooling down to −20 °C for 24 h. *n*-Butyllithium (2.5 or 1.6 mol L^−1^ solutions in hexanes, Aldrich) was used without purification. The filtering agent Celite (diatomite) was commercially acquired (Aldrich) and subjected to a drying process at 100 °C for at least five days prior to use. Elemental analyses were carried out in argon by Medac Laboratories (Chobham, Surrey, UK) using a Thermal Scientific Flash EA 1112 Series Elemental Analyzer. [TiCl_4_(thf)_2_] was prepared by a literature method.^39^

### Synthesis of [FluTi(cot)]

Fluorene (1.03 g, 6.20 mmol) was dissolved in 60 mL of a toluene/hexane mixture (1 : 1) and then cooled to −5 °C before the addition of *n*-butyllithium (6.2 mmol, 2.5 mL, 2.5 mol L^−1^ solution in hexanes). This produced a yellowish solution, which was allowed to return slowly to room temperature. After stirring for 48 hours, the resulting Li(Flu) orange suspension was used *in situ* in the subsequent reaction step, assuming a 90% yield for the deprotonation. Next, [TiCl_4_(thf)_2_] (1.86 g, 5.58 mmol) was added to 40 mL of toluene to give a bright yellow suspension that was kept stirring at room temperature for one hour. Then, an excess of cyclooctatetraene (0.87 g, 8.37 mmol) was dissolved in 30 mL of toluene and cooled to −5 °C to receive the addition of 6.7 mL (16.7 mmol) of *n*-butyllithium (2.5 mol L^−1^ solution in hexanes). The [TiCl_4_(thf)_2_] suspension was then added to the light yellow cot^2−^ solution, which immediately changed to a brown suspension. Finally, the previously prepared orange suspension of Li(Flu) was added to the reaction mixture at −5 °C, producing a reddish-brown mixture left to warm up slowly to room temperature and then stirred for 18 hours. After this period, the suspension was filtered to give a purple solid and a dark red solution. The solution was filtered again through Celite and cooled to −20 °C, yielding, after 24 hours, 0.33 g of a microcrystalline purple solid. The solid from the first filtration was then extracted with 50 mL of toluene; this produced a purple suspension filtered through Celite and cooled to −20 °C. After 24 h, this filtrate gave 0.41 g of the product as a microcrystalline purple solid. Total yield: 0.74 g, 41.8% based on the expected C_21_H_17_Ti formulation. Elemental analysis (w/w%) calculated: C: 79.51; H: 5.40. Found: C: 80.16; H: 5.60.

### [FluTi(cot)] deposition

A custom-made sublimating cell equipped with a quartz crucible was employed for molecular deposition. The air-sensitive powders were first placed in the crucible of the cell in a glove box, sealed under argon atmosphere by using a 20 cm UHV Nipple equipped with an appropriate valve, then directly installed to the sublimation chamber, and pumped to achieve 10^−8^/10^−9^ mbar base pressure. The crucible was heated to 385 K keeping in the 10^−8^ mbar pressure upon the molecular sublimation. The deposition rate was directly estimated using a quartz crystal microbalance (QCM) exposed to the sublimating molecular material. Furthermore, surface coverage was validated by STM and XPS using the Ti 2p/Au 4f ratio, which was consistent with previous findings on a similar system.^10^

### Characterization

The single-crystal X-ray diffraction analysis employed a Bruker D8 Venture diffractometer equipped with a Photon 100 area detector, a graphite monochromator, a Mo-Kα (*λ* = 0.71073 Å) radiation source, and a Kryoflex II device for low-temperature data collection. The collected data were processed with the APEX3 program,^40^ and the structure was determined by direct methods – SHELXS-97 (ref. 41 and 42) – or intrinsic phasing – SHELXT-15 – using WinGX.^43,44^

CW-EPR spectra (X-band, 9.75 GHz) were recorded on a Bruker EMX Micro spectrometer in toluene solution at room temperature (293 K) and 77 K. The samples were prepared in N_2(g)_ inside a Vacuum Atmospheres glove box. Pulsed EPR measurements at Q band (33.8 GHz) were collected on a Bruker ElexSys E580 spectrometer equipped with a dielectric ring resonator (EN 5107D2) housed in a Cryogenic Ltd cryogen-free variable temperature cryostat. High-power microwave pulses were obtained using a 10 W solid-state amplifier. During the measurements, the resonator was overcoupled to minimize ringdown following the application of microwave pulses. Electron spin echo (ESE)-detected EPR spectra were measured at *T* = 15 K using a Hahn echo sequence (π/2–*τ*–π–*τ*–echo) while sweeping the field with *τ* = 200 ns. Coherence times were measured using the Hahn echo sequence with incremented *τ*. Spin lattice relaxation times were measured using the inversion–recovery sequence π–*t*_w_–π/2–*τ*–π–*τ*–echo with incremented waiting time *t*_w_ and *τ* = 200 ns. All spectral simulations were performed with the EasySpin software package for Matlab.^45^

Scanning tunneling microscopy was performed using an Omicron VT-STM with an etched W tip. STM measurements were carried out at 35 K by fluxing liquid helium to reduce molecular mobility on the surface.

X-ray photoelectron spectroscopy XPS data were acquired *in situ* using monochromatic Al Kα radiation (*hν* = 1486.6 eV) generated by a SPECS XR-MS focus 600 source operating at a power of 100 W (13 kV and 7.7 mA). An electron analyzer, the SPECS Phoibos 150 1DLD, was mounted at 54.4° to the X-ray source, facing the sample surface for normal emission detection. Spectra were collected under normal emission conditions with a fixed pass energy set to 40 eV. CasaXPS software was employed for spectral analysis, and calibration of all XPS spectra was performed relative to the Au 4f_7/2_ signal at 83.9 eV.^46^ The deconvolution of the XPS spectra was carried out with a Hybrid Doniach Sunjic/Gaussian–Lorentzian function.

XAS and XMCD investigations were performed using the DEIMOS beamline,^47^ at the SOLEIL synchrotron facility in France. Circular polarizations were utilized, and surface sensitivity was achieved through total electron yield (TEY) detection. Samples underwent *in situ* preparation using the same sublimating cell of the in-house preparation in an identical geometric configuration at the DEIMOS beamline under ultra-high vacuum (UHV) conditions, with a base pressure of 10^−9^ mbar.

### Computational details

The vibrational spectrum of the isolated molecule was calculated using Density Functional Theory with the ORCA (v. 5.0.4) program.^48^ Geometry optimizations were conducted in the vacuum using the PBE0 functional and employing the def2-TZVP basis set.^49^ Initial coordinates were obtained from the single-crystal X-ray diffraction data. The presence of the solvent was mimicked by a conductor-like polarizable continuum model (CPCM).^16^ Frontier orbitals were calculated using the same functionals, combined with the RIJCOSX approximation^50^ and the def2/J auxiliary basis. Chemcraft was employed to draw the frontier orbitals.^51^

The *g*-tensor and the hyperfine couplings were computed on the final optimized structure in the vacuum, employing the B2PLYP functional^52^ and def2-VQZP basis set.

pDFT calculations were performed with CP2K software. An orthorhombic unit cell containing 4 Au slabs with dimensions 17.310 Å × 19.980 Å × 40.000 Å was employed. The dimensions were chosen to avoid interactions among [FluTi(cot)] replicas. The cell parameters were kept fixed throughout the optimizations. RevPBE functional,^23,53^ along with rVV10 empirical dispersion corrections,^54^ were used in all geometry optimizations. Norm-conserving Goedecker–Tetter–Hutter pseudopotentials^55^ and double zeta basis set with polarization functions DZVP-MOLOPT-SR were employed for all atoms. A Hubbard's *U* parameter of 2.2 eV, within the Dudarev implementation,^56^ was applied to the 3d orbitals of the Ti atom, as already performed for [CpTi(cot)] on Au(111).^10^ The plane-wave cut-off value was set to 450 Ry. The wavefunction convergence threshold (EPS_SCF) was set to 1.0 × 10^−6^ hartree, while the max force for the geometry optimization was set to 4.5 × 10^−3^ hartree bohr^−1^.

## Data availability

The data supporting this article have been included as part of the ESI,† including optimized geometries as XYZ files. Crystallographic data collected for [FluTi(cot)] 296 and 269 and 100 K have been deposited in the CCDC under accession numbers 2355167 and 2355169. Additional data are available from the authors upon request.

## Author contributions

Conceptualization, RS, JFS; investigation: synthesis, SW, LCC; crystallography, FSS; EPR spectroscopy, RRR, ES, MC; surface preparation and characterization, ALS, LP, NG, MM, GS; formal analysis, MB, SW; supervision, MM, AC, GS, JFS, RS; writing original draft, RS, JFS, MB, GS; writing review & editing, all authors.

## Conflicts of interest

There are no conflicts to declare.

## Supplementary Material

SC-OLF-D4SC03290J-s001

SC-OLF-D4SC03290J-s002

SC-OLF-D4SC03290J-s003

SC-OLF-D4SC03290J-s004

SC-OLF-D4SC03290J-s005

SC-OLF-D4SC03290J-s006

SC-OLF-D4SC03290J-s007

SC-OLF-D4SC03290J-s008

SC-OLF-D4SC03290J-s009

SC-OLF-D4SC03290J-s010

SC-OLF-D4SC03290J-s011

SC-OLF-D4SC03290J-s012

SC-OLF-D4SC03290J-s013

SC-OLF-D4SC03290J-s014

## References

[cit1] Goodwin C. A. P., Ortu F., Reta D., Chilton N. F., Mills D. P. (2017). Nature.

[cit2] Guo F.-S., Day B. M., Chen Y.-C., Tong M.-L., Mansikkamäki A., Layfield R. A. (2018). Science.

[cit3] Kragskow J. G. C., Mattioni A., Staab J. K., Reta D., Skelton J. M., Chilton N. F. (2023). Chem. Soc. Rev..

[cit4] Lunghi A., Sanvito S. (2019). Sci. Adv..

[cit5] de Camargo L. C., Briganti M., Santana F. S., Stinghen D., Ribeiro R. R., Nunes G. G., Soares J. F., Salvadori E., Chiesa M., Benci S., Torre R., Sorace L., Totti F., Sessoli R. (2021). Angew. Chem., Int. Ed..

[cit6] von Kugelgen S., Krzyaniak M. D., Gu M. Q., Puggioni D., Rondinelli J. M., Wasielewski M. R., Freedman D. E. (2021). J. Am. Chem. Soc..

[cit7] Ormaza M., Abufager P., Bachellier N., Robles R., Verot M., Le Bahers T., Bocquet M.-L., Lorente N., Limot L. (2015). J. Phys. Chem. Lett..

[cit8] Verlhac B., Bachellier N., Garnier L., Ormaza M., Abufager P., Robles R., Bocquet M. L., Ternes M., Lorente N., Limot L. (2019). Science.

[cit9] Czap G., Wagner P. J., Xue F., Gu L., Li J., Yao J., Wu R. Q., Ho W. (2019). Science.

[cit10] Briganti M., Serrano G., Poggini L., Sorrentino A. L., Cortigiani B., de Camargo L. C., Soares J. F., Motta A., Caneschi A., Mannini M., Totti F., Sessoli R. (2022). Nano Lett..

[cit11] Lyssenko K. A., Antipin M. Y., Ketkov S. Y. (2001). Russ. Chem. Bull..

[cit12] Samuel E., Labauze G., Vivien D. (1981). J. Chem. Soc., Dalton Trans..

[cit13] Samuel E., Labauze G., Vivien D. (1979). J. Chem. Soc., Dalton Trans..

[cit14] Eaton S. S., Harbridge J., Rinard G. A., Eaton G. R., Weber R. T. (2001). Appl. Magn. Reson..

[cit15] Lunghi A., Sanvito S. (2020). J. Phys. Chem. Lett..

[cit16] Barone V., Cossi M. (1998). J. Phys. Chem. A.

[cit17] Warner M., Din S., Tupitsyn I. S., Morley G. W., Stoneham A. M., Gardener J. A., Wu Z., Fisher A. J., Heutz S., Kay C. W. M., Aeppli G. (2013). Nature.

[cit18] Graham M. J., Yu C. J., Krzyaniak M. D., Wasielewski M. R., Freedman D. E. (2017). J. Am. Chem. Soc..

[cit19] Groenenboom C. J., Sawatzky G., de Liefde Meijer H. J., Jellinek F. (1974). J. Organomet. Chem..

[cit20] Jaeger D., Patscheider J. (2013). Surf. Sci. Spectra.

[cit21] Jaeger D., Patscheider J. (2012). J. Electron Spectrosc. Relat. Phenom..

[cit22] Porte L., Roux L., Hanus J. (1983). Phys. Rev. B: Condens. Matter Mater. Phys..

[cit23] Perdew J. P., Burke K., Ernzerhof M. (1996). Phys. Rev. Lett..

[cit24] Tao J. M., Perdew J. P., Staroverov V. N., Scuseria G. E. (2003). Phys. Rev. Lett..

[cit25] Cho F. H., Park J., Oh S., Yu J., Jeong Y., Colazzo L., Spree L., Hommel C., Ardavan A., Boero G., Donati F. (2024). Rev. Sci. Instrum..

[cit26] Li Y., Weng Y., Yin X., Yu X., Kumar S. R. S., Wehbe N., Wu H., Alshareef H. N., Pennycook S. J., Breese M. B. H., Chen J., Dong S., Wu T. (2018). Adv. Funct. Mater..

[cit27] Chen S. C., Sung K. Y., Tzeng W. Y., Wu K. H., Juang J. Y., Uen T. M., Luo C. W., Lin J. Y., Kobayashi T., Kuo H. C. (2013). J. Phys. D: Appl. Phys..

[cit28] Yan D., Topsakal M., Selcuk S., Lyons J. L., Zhang W., Wu Q., Waluyo I., Stavitski E., Attenkofer K., Yoo S., Hybertsen M. S., Lu D., Stacchiola D. J., Liu M. (2019). Nano Lett..

[cit29] Ohtomo A., Muller D. A., Grazul J. L., Hwang H. Y. (2002). Nature.

[cit30] Thakur H., Thakur P., Kumar R., Brookes N. B., Sharma K. K., Singh A. P., Kumar Y., Gautam S., Chae K. H. (2011). Appl. Phys. Lett..

[cit31] Tesi L., Lucaccini E., Cimatti I., Perfetti M., Mannini M., Atzori M., Morra E., Chiesa M., Caneschi A., Sorace L., Sessoli R. (2016). Chem. Sci..

[cit32] Bachellier N., Ormaza M., Faraggi M., Verlhac B., Verot M., Le Bahers T., Bocquet M. L., Limot L. (2016). Phys. Rev. B.

[cit33] Willke P., Bilgeri T., Zhang X., Wang Y., Wolf C., Aubin H., Heinrich A., Choi T. (2021). ACS Nano.

[cit34] Yang K., Paul W., Phark S. H., Willke P., Bae Y., Choi T., Esat T., Ardavan A., Heinrich A. J., Lutz C. P. (2019). Science.

[cit35] Wang Y., Chen Y., Bui H. T., Wolf C., Haze M., Mier C., Kim J., Choi D.-J., Lutz C. P., Bae Y., Phark S.-h., Heinrich A. J. (2023). Science.

[cit36] Heinrich A. J., Oliver W. D., Vandersypen L. M. K., Ardavan A., Sessoli R., Loss D., Jayich A. B., Fernandez-Rossier J., Laucht A., Morello A. (2021). Nat. Nanotechnol..

[cit37] Mahieu N., Piątkowski J., Simler T., Nocton G. (2023). Chem. Sci..

[cit38] Perrin D. D. and Armarego W. L., Purification of Laboratory Chemicals, Butterworth-Heinemann, Oxford, 3rd edn, 1997

[cit39] Sun D., Liu W., Qiu M., Zhang Y., Li Z. (2015). Chem. Commun..

[cit40] APEX3, SAINT and SADABS software, 2016

[cit41] Sheldrick G. (2015). Acta Crystallogr., Sect. C: Struct. Chem..

[cit42] SHELX-97, an integrated system for solving and refining crystal structure from diffraction data, 1997

[cit43] Farrugia L. (1999). J. Appl. Crystallogr..

[cit44] Farrugia L. (2012). J. Appl. Crystallogr..

[cit45] Stoll S., Schweiger A. (2006). J. Magn. Reson..

[cit46] Heister K., Zharnikov M., Grunze M., Johansson L. S. O. (2001). J. Phys. Chem. B.

[cit47] Ohresser P., Otero E., Choueikani F., Chen K., Stanescu S., Deschamps F., Moreno T., Polack F., Lagarde B., Daguerre J. P., Marteau F., Scheurer F., Joly L., Kappler J. P., Muller B., Bunau O., Sainctavit P. (2014). Rev. Sci. Instrum..

[cit48] Neese F. (2018). Wiley Interdiscip. Rev.: Comput. Mol. Sci..

[cit49] Weigend F., Ahlrichs R. (2005). Phys. Chem. Chem. Phys..

[cit50] Neese F., Wennmohs F., Hansen A., Becker U. (2009). Chem. Phys..

[cit51] Chemcraft - graphical software for visualization of quantum chemistry computations. Version 1.8, build 682, 2020, https://www.chemcraftprog.com

[cit52] Grimme S. (2006). J. Chem. Phys..

[cit53] Zhang Y., Yang W. (1998). Phys. Rev. Lett..

[cit54] Sabatini R., Gorni T., de Gironcoli S. (2013). Phys. Rev. B: Condens. Matter Mater. Phys..

[cit55] Goedecker S., Teter M., Hutter J. (1996). Phys. Rev. B: Condens. Matter Mater. Phys..

[cit56] Dudarev S. L., Botton G. A., Savrasov S. Y., Humphreys C. J., Sutton A. P. (1998). Phys. Rev. B: Condens. Matter Mater. Phys..

