# Supplementary material for: (η8-Cyclooctatetraene)(η5-fluorenyl)titanium: a processable molecular spin qubit with optimized control of the molecule–substrate interface
Source: Chem Sci. 2024 Aug 9;15(35):14390–8. doi: 10.1039/d4sc03290j (PMC11325857; doi:10.1039/d4sc03290j)
Supplement: SC-015-D4SC03290J-s001 [file SC-015-D4SC03290J-s001.pdf]

Supplementary Information (SI) for Chemical Science.
This journal is © The Royal Society of Chemistry 2024

(η^8-Cyclooctatetraene)(η^5-fluorenyl)titanium: a processable molecular spin qubit with optimized control of the molecule-substrate interface

Sarita Wisbeck,[a§] Andrea Luigi Sorrentino,[b§] Francielli S. Santana,[a] Luana C. de Camargo,[a] Ronny R. Ribeiro,[a] Enrico Salvadori,[c] Mario Chiesa,[c] Niccolò Giaconi,[b] Andrea Caneschi,[d] Matteo Mannini,[b] Lorenzo Poggini,[e] Matteo Briganti,[b]* Giulia Serrano,[d]* Jaísa F. Soares,[a]* Roberta Sessoli[b,e]*

Electronically Supplementary Information

[a.] Department of Chemistry, Federal University of Paraná, Centro Politécnico, Jardim das Américas, 81530-900 Curitiba-PR, Brazil
[b.] Department of Chemistry "U. Schiff" (DICUS) and INSTM UdR Firenze, University of Florence, Via della Lastruccia 3-13, 50019 Sesto Fiorentino, Italy
[c.] Department of Chemistry, University of Turin, Via Giuria 7, 10125 Torino, Italy
[d.] Department of Industrial Engineering (DIEF) and INSTM Research Unit, University of Florence, Florence, Italy
[e.] Institute for Chemistry of OrganoMetallic Compounds (ICCOM-CNR), Via Madonna del Piano, 50019 Sesto Fiorentino, Italy

e-mail: roberta.sessoli@unifi.it; jaisa.soares@ufpr.br; giulia.serrano@unifi.it, matteo.briganti@unifi.it

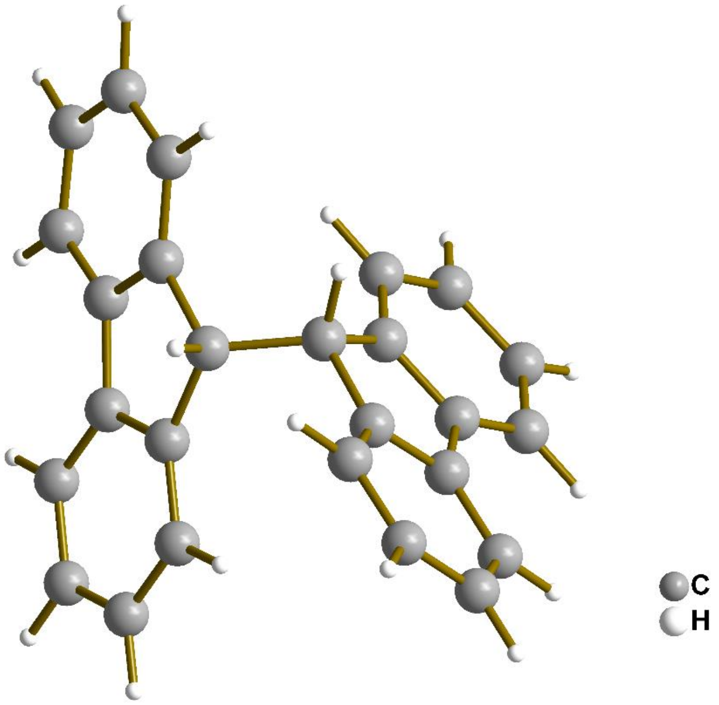

Figure S1. Representation of the molecular structure of 9,9'-bifluorenyl.

This dimer had been obtained upon oxidation of Flu^{-} (fluorenide) by organic electron acceptors,[1,2] transition metal halide radical coupling,[3,4] or reductive coupling of 9-bromofluorene.[5] In our work, colorless crystals of 9,9'-bifluorenyl were isolated from the reaction mixture when fluorenyl lithium, LiFlu, was mixed with a suspension of [$TiCl_4$(thf)$_2$] (thf = tetrahydrofuran) in toluene before the addition of cyclooctatetraene and excess of n-butyllithium ($LiBu^n$). In this case, the titanium(IV) precursor oxidized Flu^{-} and led to unwanted dimerization with the concomitant reduction of titanium(IV) to titanium(III). The identity of the colorless product (9,9'-bifluorenyl) was verified by single-crystal X-ray diffraction analysis. This redox reaction prevented the formation of [FluTi(cot)], which was only obtained in reasonable yield after the order of addition was changed to allow a prior reaction of [$TiCl_4$(thf)$_2$] with Li_2(cot) and one additional equivalent of LiBu, before the addition of LiFlu, as described in the Experimental (main text).

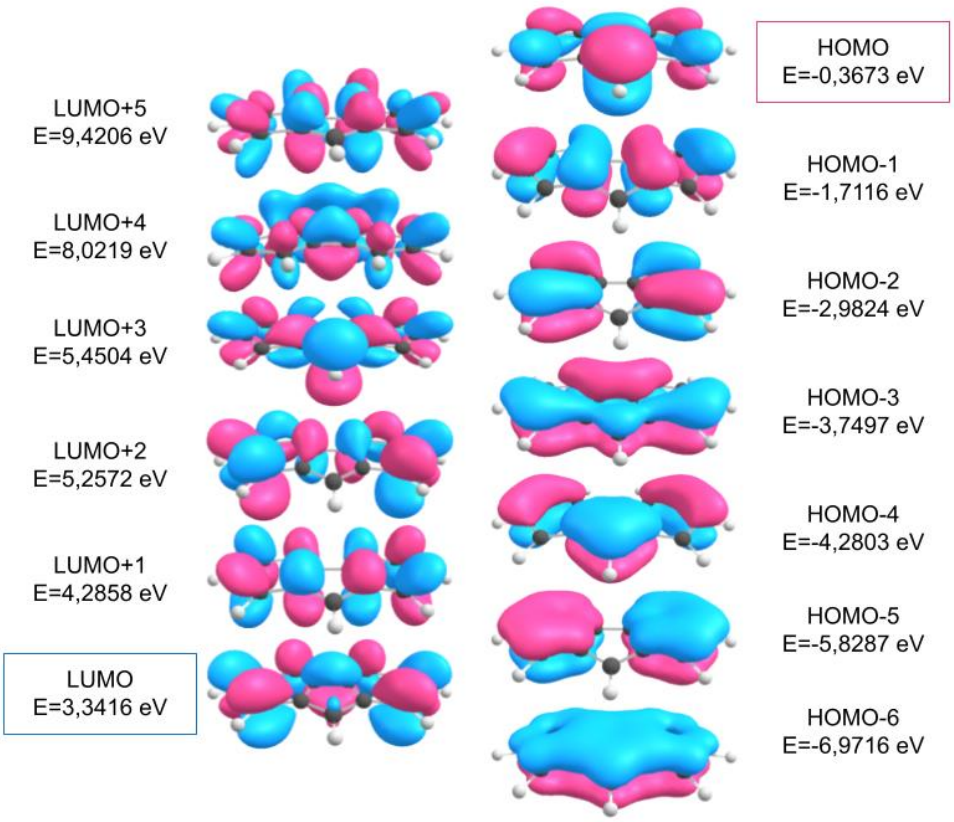

Figure S2: Energies and contour surfaces (30%) of the frontier molecular orbitals in Flu^{-}. The atomic coordinates were optimized in the vacuum, starting from the atomic coordinates of the fluorenyl ligand in the crystal structure of [FluTi(cot)].

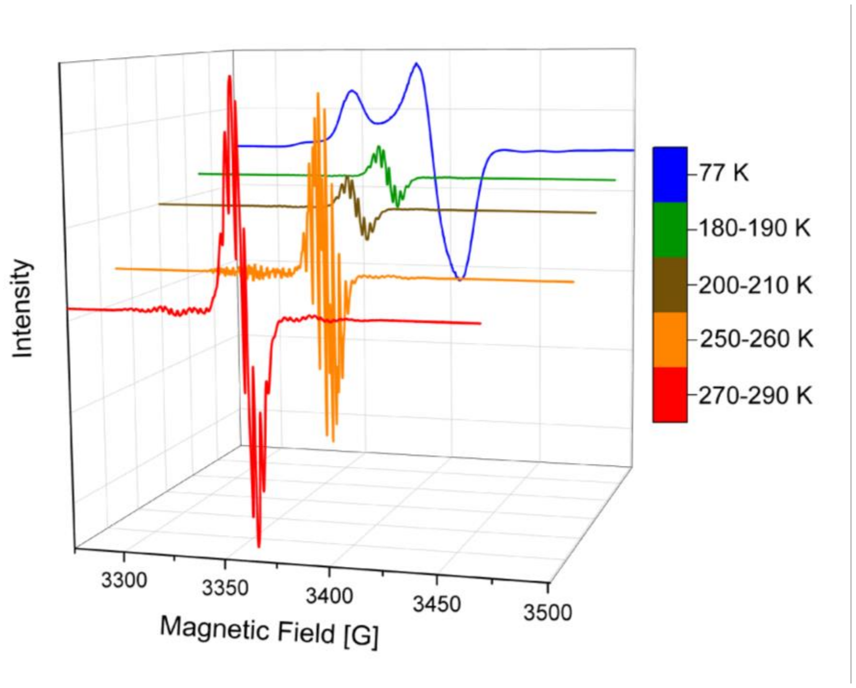

Figure S3. Selected X-band, CW-EPR spectra of [FluTi(cot)] recorded during slow thawing of a 1.0 mmol L^{-1} solution of [FluTi(cot)] in toluene.

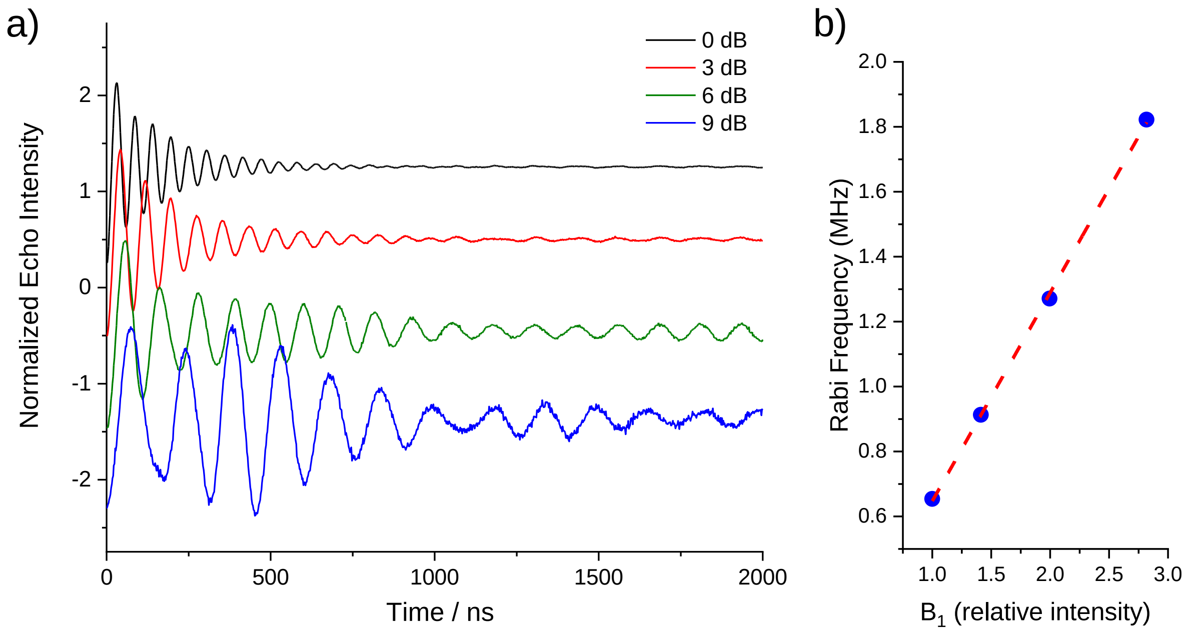

Figure S4. Nutation experiments performed on a 0.5 mmol L^{-1} solution of [FluTi(cot)] in toluene-d_8 at T = 15 K and Q-band frequency for four attenuations; a) Normalized echo intensity; b) Extracted Rabi frequency (blue dots) as a function of the intensity of the microwave radiation, well reproduced by assuming linear dependence (red line).

Table S1. Crystal and structure refinement data for [(η^8-C_8H_8)Ti(η^5-$C_{13}H_9$), [FluTi(cot)] (data collection carried out at two different temperatures, 100(2) and 296(2) K).

Elemental formula	C21 H17 Ti	C21 H17 Ti
Formula weight	317.24	317.24
CCDC deposition number	2355169	2355167
Temperature of data collection	100(2) K	296(2) K
Wavelength	0.71073 Å	0.71073 Å
Crystal system, space group	Orthorhombic, *P*nma	Orthorhombic, *P*nma
Unit cell dimensions	*a* = 8.3693(5) Å<br>*b* = 15.9237(8) Å<br>*c* = 11.3545(6) Å<br>α = β = γ = 90°	*a* = 8.5539(5) Å<br>*b* = 15.9444(10) Å<br>*c* = 11.4097(6) Å<br>α = β = γ = 90°
Volume	1513.22(14) $Å^3$	1556.13(16) $Å^3$
Z, Calculated density	4, 1.393 Mg/m^3	4, 1.354 Mg/m^3
F(000)	660	660
Absorption coefficient	0.558 mm^{-1}	0.543 mm^{-1}
Crystal color, shape	violet, rhomb	violet, rhomb
Crystal size	0.465 x 0.274 x 0.160 mm	0.319 x 0.241 x 0.104 mm
Theta range for data collection	2.2 to 27.5 °	3.0 to 27.0 °
Limiting indices	$-10 \le h \le 10$, $-20 \le k \le 20$, $-14 \le l \le 14$	$-11 \le h \le 11$, $-20 \le k \le 20$, $-14 \le l \le 14$
Completeness to θ = 25.2 °	99.7 %	99.7 %
Absorption correction	Semi-empirical from equivalents	Semi-empirical from equivalents
Max. and min. transmission	0.7456 and 0.7069	0.7456 and 0.7010
Reflections collected / unique	33264 / 1805 [R_{int} = 0.029]	59572 / 1856 [R_{int} = 0.051]
No. of 'observed' reflections ($I > 2\sigma_I$)	1679	1576
Data / restraints / parameters	1805 / 0 / 144	1856 / 9 / 143
Goodness-of-fit on F^2	1.048	1.073
Final R indices ('observed' data)	R_1 = 0.029, wR_2 = 0.070 [(1)]	R_1 = 0.039, wR_2 = 0.093 [(2)]
Final R indices (all data)	R_1 = 0.032, wR_2 = 0.071 [(1)]	R_1 = 0.051, wR_2 = 0.101 [(2)]
Extinction coefficient	0.0096(14)	n/a
Largest difference between peak and hole	0.85 and −0.48 e $Å^{-3}$	0.27 and −0.43 e $Å^{-3}$
Location of largest difference peak	0.80 Å from the Ti atom	0.63 Å from the C2 atom

[(1)] Reflections weighted $w = [\sigma^2(F_o^2)+(0.0276P)^2+1.3633*P]^{-1}$, where $P=(F_o^2+2F_c^2)/3$

[(2)] Reflections weighted $w = [\sigma^2(F_o^2)+(0.0412P)^2+0.9777*P]^{-1}$, where $P=(F_o^2+2F_c^2)/3$.

Table S2: Molecular dimensions involving the Ti atom. Bond lengths are in Å, and angles in degrees (data collection at 100(2) K).

Ti−C1	2.3413(18)	Ti−C2	2.3836(12)
Ti−C2^{i}	2.3836(12)	Ti−C7	2.4008(12)
Ti−C8	2.301(2)	Ti−C9	2.3098(15)
Ti−C9^{i}	2.3098(15)	Ti−C10	2.3141(15)
Ti−C10^{i}	2.3140(15)	Ti−C11	2.3187(15)
Ti−C11^{i}	2.3187(15)	Ti−C12	2.323(2)
C1−Ti−C2	35.11(4)	C1−Ti−C2^{i}	35.11(4)
C2−Ti−C2^{i}	57.53(6)	C1−Ti−C7	59.05(5)
C2−Ti−C7	35.03(4)	C2^{i}−Ti−C7	58.12(4)
C8−Ti−C1	95.79(7)	C8−Ti−C2	113.32(6)
C8−Ti−C2^{i}	113.32(6)	C8−Ti−C7	148.26(6)
C8−Ti−C9	35.35(5)	C8−Ti−C9^{i}	35.35(5)
C8−Ti−C10	68.11(6)	C8−Ti−C10^{i}	68.11(6)
C8−Ti−C11	93.99(6)	C8−Ti−C11^{i}	93.99(6)
C8−Ti−C12	104.57(8)	C9−Ti−C1	103.17(6)
C9^{i}−Ti−C1	103.17(6)	C9−Ti−C2	134.97(6)
C9^{i}−Ti−C2	99.67(5)	C9−Ti−C2^{i}	99.67(5)
C9^{i}−Ti−C2^{i}	134.97(6)	C9−Ti−C7	157.79(5)
C9^{i}−Ti−C7	125.99(6)	C9−Ti−C9^{i}	68.07(10)
C9−Ti−C10	35.25(7)	C9−Ti−C10^{i}	93.72(7)
C9^{i}−Ti−C10^{i}	35.25(7)	C9^{i}−Ti−C10	93.72(6)
C9−Ti−C11	67.92(7)	C9−Ti−C11^{i}	104.31(6)
C9^{i}−Ti−C11	104.30(6)	C9^{i}−Ti−C11^{i}	67.92(7)
C9−Ti−C12	93.64(6)	C9^{i}−Ti−C12	93.64(6)
C10−Ti−C1	121.72(5)	C10^{i}−Ti−C1	121.72(5)
C10−Ti−C2	155.55(5)	C10^{i}−Ti−C2	98.76(5)
C10−Ti−C2^{i}	98.76(5)	C10^{i}−Ti−C2^{i}	155.55(5)
C10−Ti−C7	140.02(5)	C10^{i}−Ti−C7	106.89(5)
C10^{i}−Ti−C10	103.92(8)	C10−Ti−C11	35.05(7)
C10−Ti−C11^{i}	93.33(6)	C10^{i}−Ti−C11	93.33(6)
C10^{i}−Ti−C11^{i}	35.05(7)	C10−Ti−C12	67.60(6)
C10^{i}−Ti−C12	67.59(6)	C11−Ti−C1	144.75(5)
C11^{i}−Ti−C1	144.75(5)	C11−Ti−C2	152.60(5)
C11−Ti−C2^{i}	110.62(5)	C11^{i}−Ti−C2	110.62(5)
C11^{i}−Ti−C2^{i}	152.60(5)	C11−Ti−C7	117.74(5)
C11^{i}−Ti−C7	97.42(5)	C11^{i}−Ti−C11	67.64(10)
C11−Ti−C12	35.04(5)	C11^{i}−Ti−C12	35.04(5)
C12−Ti−C1	159.65(7)	C12−Ti−C2	131.11(5)
C12−Ti−C2^{i}	131.11(5)	C12−Ti−C7	101.71(6)
C2−C1−Ti	74.07(9)	C2^{i}−C1−Ti	74.07(9)
C2−C7−Ti	71.83(7)	C3−C2−Ti	119.91(9)
C6−C7−Ti	121.53(9)	C7−C2−Ti	73.14(7)
C7^{i}−C7−Ti	72.42(3)	C8−C9−Ti	72.00(10)
C9−C8−Ti	72.66(10)	C9^{i}−C8−Ti	72.66(10)
C9−C10−Ti	72.21(9)	C10−C9−Ti	72.54(9)
C10−C11−Ti	72.29(9)	C11−C10−Ti	72.66(9)
C11−C12−Ti	72.33(10)	C11^{i}−C12−Ti	72.33(10)
C12−C11−Ti	72.63(10)	Ti−C1−H1	121.5(15)
Ti−C8−H8	129.8(18)	Ti−C9−H9	129.3(15)
Ti−C10−H10	129.7(14)	Ti−C11−H11	131.9(13)
Ti−C12−H12	132.1(19)		

Symmetry transformations used to generate equivalent atoms: (i) x, −y+1/2, z.

Table S3: Molecular dimensions involving the Ti atom. Bond lengths are in Å, angles in degrees (data collection at 296(2) K).

Ti−C1	2.343(3)	Ti−C2	2.3865(18)
Ti−C2^{i}	2.3865(18)	Ti−C7^{i}	2.4005(17)
Ti−C8	2.274(4)	Ti−C9	2.276(3)
Ti−C9^{i}	2.276(3)	Ti−C10	2.284(3)
Ti−C10^{i}	2.284(3)	Ti−C11	2.289(3)
Ti−C11^{i}	2.289(3)	Ti−C12	2.286(4)
C1−Ti−C2	34.99(6)	C1−Ti−C2^{i}	34.99(6)
C2−Ti−C2^{i}	57.48(10)	C1−Ti−C7^{i}	58.59(8)
C2^{i}−Ti−C7^{i}	34.84(6)	C2−Ti−C7^{i}	57.83(6)
C8−Ti−C1	97.68(14)	C8−Ti−C2	114.78(11)
C8−Ti−C2^{i}	114.78(11)	C8−Ti−C7^{i}	149.49(11)
C8−Ti−C9	35.24(18)	C8−Ti−C9^{i}	35.24(18)
C8−Ti−C10	67.67(18)	C8−Ti−C10^{i}	67.67(18)
C8−Ti−C11	92.74(15)	C8−Ti−C11^{i}	92.74(15)
C8−Ti−C12	102.74(16)	C9−Ti−C1	104.82(12)
C9^{i}−Ti−C1	104.82(12)	C9−Ti−C2	136.42(14)
C9^{i}−Ti−C2	100.84(11)	C9−Ti−C2^{i}	100.84(11)
C9^{i}−Ti−C2^{i}	136.42(14)	C9^{i}−Ti−C7^{i}	158.67(11)
C9−Ti−C7^{i}	126.65(14)	C9−Ti−C9^{i}	67.8(3)
C9−Ti−C10	35.04(19)	C9−Ti−C10^{i}	92.9(2)
C9^{i}−Ti−C10^{i}	35.04(19)	C9^{i}−Ti−C10	92.9(2)
C9−Ti−C11	67.1(2)	C9−Ti−C11^{i}	102.63(11)
C9^{i}−Ti−C11	102.63(11)	C9^{i}−Ti−C11^{i}	67.1(2)
C9−Ti−C12	92.05(15)	C9^{i}−Ti−C12	92.05(15)
C10−Ti−C1	122.99(14)	C10^{i}−Ti−C1	122.99(14)
C10−Ti−C2	156.59(12)	C10^{i}−Ti−C2	99.69(10)
C10−Ti−C2^{i}	99.69(10)	C10^{i}−Ti−C2^{i}	156.59(12)
C10^{i}−Ti−C7^{i}	139.92(16)	C10−Ti−C7^{i}	107.32(13)
C10^{i}−Ti−C10	102.40(17)	C10−Ti−C11	34.59(19)
C10−Ti−C11^{i}	91.4(2)	C10^{i}−Ti−C11	91.4(2)
C10^{i}−Ti−C11^{i}	34.59(19)	C10−Ti−C12	66.28(18)
C10^{i}−Ti−C12	66.28(18)	C11−Ti−C1	145.46(13)
C11^{i}−Ti−C1	145.46(13)	C11−Ti−C2	152.46(12)
C11−Ti−C2^{i}	111.31(12)	C11^{i}−Ti−C2	111.31(12)
C11^{i}−Ti−C2^{i}	152.46(12)	C11^{i}−Ti−C7^{i}	117.71(13)
C11−Ti−C7^{i}	97.98(9)	C11^{i}−Ti−C11	65.9(3)
C11−Ti−C12	34.15(17)	C11^{i}−Ti−C12	34.15(17)
C12−Ti−C1	159.58(13)	C12−Ti−C2	131.21(9)
C12−Ti−C2^{i}	131.21(9)	C12−Ti−C7^{i}	102.10(11)
C2−C1−Ti	74.20(13)	C2^{i}−C1−Ti	74.20(13)
C2−C7−Ti	72.05(10)	C3−C2−Ti	120.05(14)
C6−C7−Ti	121.05(13)	C7−C2−Ti	73.11(10)
C7^{i}−C7−Ti	72.55(5)	C8−C9−Ti	72.3(2)
C9−C8−Ti	72.5(2)	C9^{i}−C8−Ti	72.5(2)
C9−C10−Ti	72.18(19)	C10−C9−Ti	72.78(19)
C10−C11−Ti	72.5(2)	C11−C10−Ti	72.9(2)
C11−C12−Ti	73.1(2)	C11^{i}−C12−Ti	73.1(2)
C12−C11−Ti	72.8(2)	Ti−C1−H1	123(2)
Ti−C8−H8	130(3)	Ti−C9−H9	124(3)
Ti−C10−H10	129(3)	Ti−C11−H11	128(3)
Ti−C12−H12	132(3)		

Symmetry transformations used to generate equivalent atoms: (i) x, −y+1/2, z.

Table S4. Computed hyperfine coupling tensors of Ti and all the hydrogen atoms within DFT/B2PLYP approach. The hydrogen's label is the same as shown in Figure 1.

Atom	A(iso)	A_x	A_y	A_z
Ti (I = 47)	39.1258	58.17	55.16	4.05
H1 (Flu)	4.1961	9.55	1.72	1.32
H3 (Flu)	0.4457	-0.77	3.11	-1.00
H4 (Flu)	-0.6399	-1.59	0.90	-1.23
H5 (Flu)	0.2188	-0.34	1.49	-0.49
H6 (Flu)	-0.4964	2.20	-1.94	-1.76
H3' (Flu)	0.4454	-0.77	3.11	-1.01
H4' (Flu)	-0.6400	-1.59	0.90	-1.23
H5' (Flu)	0.2189	-0.34	1.49	-0.49
H6' (Flu)	-0.4961	2.21	-1.94	-1.76
H8 (cot)	8.5093	13.32	6.77	5.45
H9 (cot)	8.2419	6.44	13.10	5.19
H10 (cot)	7.2943	5.44	12.17	4.27
H11 (cot)	6.8693	11.68	5.04	3.89
H12 (cot)	7.0014	11.71	5.28	4.02
H9' (cot)	8.2424	6.44	13.10	5.19
H10' (cot)	7.2940	5.44	12.17	4.27
H11' (cot)	6.8691	11.68	5.04	3.89

Estimation of the nuclear spin contribution to T_m

The coherence times at the single molecule level were estimated employing the computed values of the hyperfine coupling constants. The hydrogen nuclei contributions to the coherence time were computed according to previous works.[6-8] Within this approach, $T_m = \hbar\Delta/(E_n)^2$, where E_n is the nuclear contribution to the echo linewidth, and Δ is the value of the gap between the two qubit states (33.3 GHz here). E_n can be computed with the formula:

$$E_n^2 = \sum_k \frac{\left(\omega_k^{\parallel}\right)^2 I_k(I_k+1)}{3}$$

where ω_k is the difference between the interaction energies of the electronic spin with the kth active nuclear spin when the electronic spin is in two different states.

Table S5. Computed vibrational spectrum in the vacuum and in implicit solvent.

Vacuum		Implicit solvent (hexane)	
**Number**	**Frequency (cm^{-1})**	**Number**	**Frequency (cm^{-1})**
6	31.74	6	31.52
7	87.15	7	83.15
8	90.17	8	84.74
9	102.06	9	101.40
10	129.36	10	129.60
11	130.88	11	130.13
12	156.01	12	154.88
13	219.83	13	219.26
14	220.36	14	220.55
15	236.44	15	234.05
16	245.29	16	241.27
17	308.65	17	309.30
18	333.94	18	333.63
19	362.44	19	361.48
20	368.99	20	368.60
21	392.39	21	391.85
22	400.29	22	399.85
23	400.43	23	400.08
24	433.25	24	432.74
25	458.17	25	456.85
26	481.46	26	482.26

Table S6. Pictorial representation of the computed normal modes in the vacuum. The frequencies' character is classified within approximate C_s point group symmetry.

Frequency	Character	Composition
31.74	antisymmetric	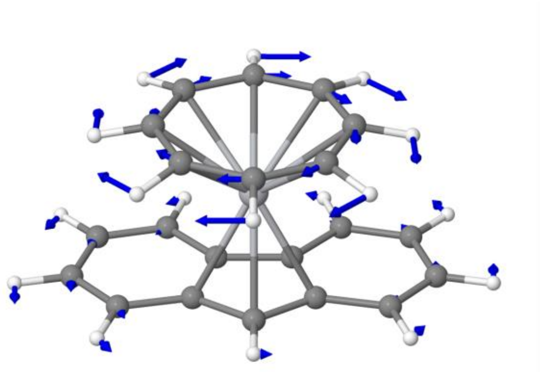
87.15	antisymmetric	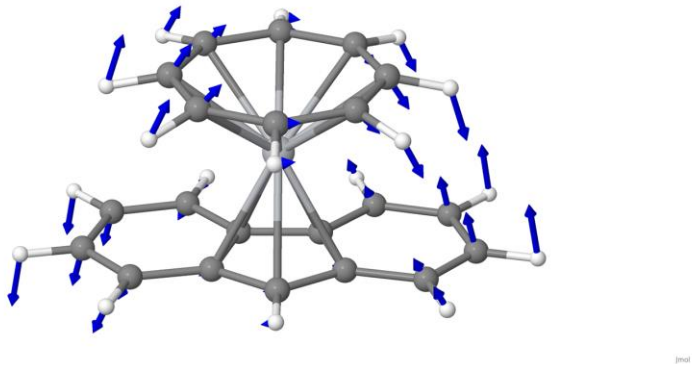
90.17	symmetric	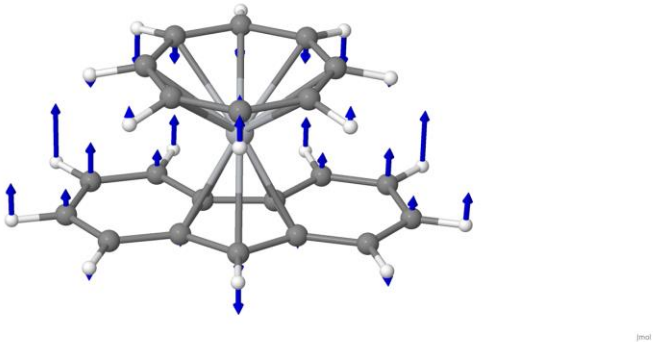

Table S6 (continuation).

Frequency	Character	Composition
102.06	symmetric	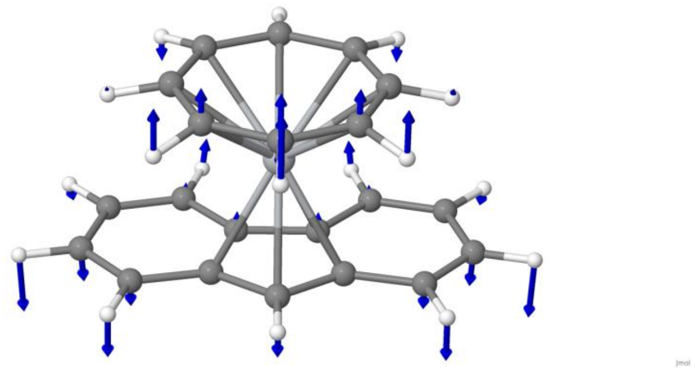
129.36	symmetric	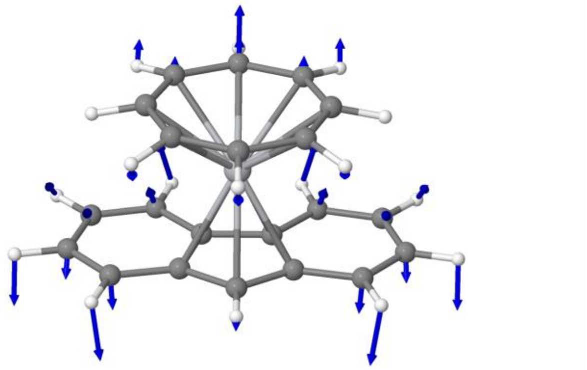
130.88	antisymmetric	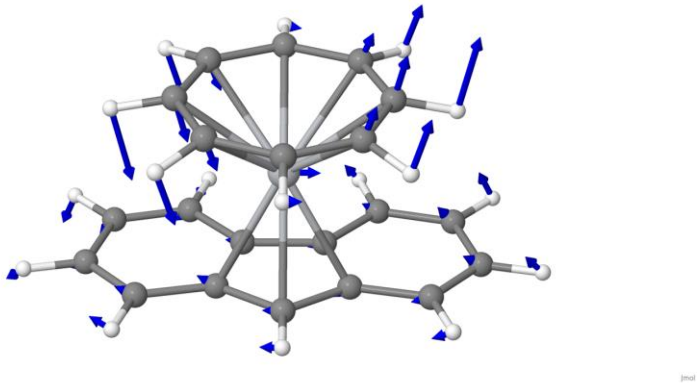

Table S7. *Lying*$_1$ configuration: Mulliken Spin density for 'Top' and 'Bridge' sites and for different U values combined with revPBE functional.

**U value**	**Adsorption site**	
	Bridge 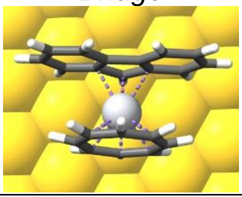	*Top* 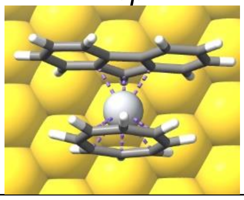
2.2 eV	0.07712	0.017035
3.0 eV	0.040916	-0.010603

Table S8. Adsorption energies of the four examined configurations computed with three different density functionals.

**Adsorption Energy (kcal/mol)**			
Orientation	***Density Functional***		
	Revised PBE	*Original PBE*	*TPSS*
Standing$_{Flu}$	-32.13	-48.70	-48.20
Standing$_{cot}$	-24.75	-36.83	-37.21
Lying$_1$	-24.44	-36.32	-28.52
Lying$_2$	-23.87	-35.51	-27.93

References

1 R. D. Guthrie, D. P. Wesley, G. W. Pendygraft and A. T. Young, *J. Am. Chem. Soc.*, 1976, **98**, 5870-5877.
2 R. D. Guthrie, C. Hartmann, R. Neill and D. E. Nutter, *The Journal of Organic Chemistry*, 1987, **52**, 736-740.
3 S. Y. Knjazhanski, G. Moreno, G. Cadenas, V. K. Belsky and B. M. Bulychev, *Tetrahedron*, 1999, **55**, 1639-1646.
4 M. H. Al-Afyouni, T. A. Huang, F. Hung-Low and C. A. Bradley, *Tetrahedron Lett.*, 2011, **52**, 3261-3265.
5 V. S. Sridevi, W. K. Leong and Y. Zhu, *Organometallics*, 2006, **25**, 283-288.
6 P. C. E. Stamp and I. S. Tupitsyn, *Chem. Phys.*, 2004, **296**, 281-293.
7 M. Warner, S. Din, I. S. Tupitsyn, G. W. Morley, A. M. Stoneham, J. A. Gardener, Z. Wu, A. J. Fisher, S. Heutz, C. W. M. Kay and G. Aeppli, *Nature*, 2013, **503**, 504-508.
8 L. Escalera-Moreno, A. Gaita-Ariño and E. Coronado, *Phys. Rev. B*, 2019, **100**, 064405.
